# Artificial intelligence-driven mobile interpretation of a semi-quantitative cryptococcal antigen lateral flow assay

**DOI:** 10.1186/s43008-024-00158-5

**Published:** 2024-08-30

**Authors:** David Bermejo-Peláez, Ana Alastruey-Izquierdo, Narda Medina, Daniel Capellán-Martín, Oscar Bonilla, Miguel Luengo-Oroz, Juan Luis Rodríguez-Tudela

**Affiliations:** 1 Spotlab, Madrid, Spain; 2 Mycology Reference Laboratory, National Center for Microbiology, Instituto de Salud Carlos III, Madrid, Spain; 3 Center for Biomedical Research in Network in Infectious Diseases (CIBERINFEC-CB21/13/00105), Instituto de Salud Carlos III, Madrid, Spain; 4 Asociación de Salud Integral, Guatemala City, Guatemala; 5 Clínica Familiar “Luis Ángel García”, Hospital General San Juan de Dios, Guatemala City, Guatemala; 6 Global Action for Fungal Infections, Geneva, Switzerland

**Keywords:** Cryptococcosis, Lateral flow assay (LFA), Artificial intelligence (AI), Smartphone, Semiquantitative assay, Antigen quantification

## Abstract

**Objectives:**

Cryptococcosis remains a severe global health concern, underscoring the urgent need for rapid and reliable diagnostic solutions. Point-of-care tests (POCTs), such as the cryptococcal antigen semi-quantitative (CrAgSQ) lateral flow assay (LFA), offer promise in addressing this challenge. However, their subjective interpretation poses a limitation. Our objectives encompass the development and validation of a digital platform based on Artificial Intelligence (AI), assessing its semi-quantitative LFA interpretation performance, and exploring its potential to quantify CrAg concentrations directly from LFA images.

**Methods:**

We tested 53 cryptococcal antigen (CrAg) concentrations spanning from 0 to 5000 ng/ml. A total of 318 CrAgSQ LFAs were inoculated and systematically photographed twice, employing two distinct smartphones, resulting in a dataset of 1272 images. We developed an AI algorithm designed for the automated interpretation of CrAgSQ LFAs. Concurrently, we explored the relationship between quantified test line intensities and CrAg concentrations.

**Results:**

Our algorithm surpasses visual reading in sensitivity, and shows fewer discrepancies (p < 0.0001). The system exhibited capability of predicting CrAg concentrations exclusively based on a photograph of the LFA (Pearson correlation coefficient of 0.85).

**Conclusions:**

This technology's adaptability for various LFAs suggests broader applications. AI-driven interpretations have transformative potential, revolutionizing cryptococcosis diagnosis, offering standardized, reliable, and efficient POCT results.

**Supplementary Information:**

The online version contains supplementary material available at 10.1186/s43008-024-00158-5.

## Introduction

Cryptococcosis is a severe opportunistic fungal infection that primarily affects people with HIV (Antinori 2013; Firacative et al. 2021). *Cryptococcus neoformans*, the main etiologic agent of Cryptococcosis, has been classified by the World Health Organization (WHO) as a critical priority pathogen (World Health Organization 2022b). In 2020, there were 680,000 HIV-related deaths worldwide, of which 19% were attributed to cryptococcosis. Although the incidence of the disease varies by geographic location, it is estimated that more than 150,000 cases of cryptococcal meningitis occur annually worldwide (Rajasingham et al. 2022; World Health Organization 2018). Latin America has an incidence of 5300 cases of *Cryptococcus*-associated meningitis per year, and in a prospective cohort study conducted in Guatemala among HIV patients, the overall incidence of cryptococcal disease was 8.7% in patients with < 200 CD4/mm3 (Firacative et al. 2018; Medina et al. 2021). Early detection and proper treatment are fundamental to reducing morbidity and mortality.

The WHO guidelines for the diagnosis, prevention, and management of cryptococcal infection in HIV-infected patients recommend screening of cryptococcal antigen in all patients with a CD4 cell count of less than 200 (World Health Organization 2018, 2022a). For disease screening, immunochromatographic tests such as lateral flow assays (LFA) have been shown to be optimal due to their adaptability to resource-limited settings and ability to be performed at the point-of-care (POC). In addition, cryptococcal antigen (CrAg) LFA test offer results in around 10 min, with sensitivity and specificity of > 99% and 98%, respectively (Tang et al. 2016).

IMMY, a diagnostic company (https://www.immy.com), has developed two tests for detecting CrAg: the CrAg® LFA (qualitative) and the CrAgSQ LFA (semi-quantitative in one step). Currently, the qualitative test is the most widely used worldwide, providing a binary outcome of either positive or negative. Positive results from this test necessitate a lumbar puncture to rule out cryptococcal meningitis. However, this recommendation is not universally implemented due to various reasons, leading to the omission of this diagnostic procedure in many cases with positive CrAg results. A more comprehensive risk assessment for patients with a positive CrAg test can reinforce the importance of lumbar puncture for the diagnosis or exclusion of cryptococcal meningitis. Moreover, in cases of high cryptococcal antigen concentrations, the qualitative test may produce false negatives due to the postzone effect which is when there is an excess of antigen relative to antibody leading to a decrease in the visible reaction between the antigen and the antibody. This effect is observed in immunoassays, such as precipitation or agglutination reactions, and can lead to false-negative results. Beyond simply indicating positivity or negativity, quantifying CrAg concentrations holds significant importance. Studies have linked higher CrAg titers to increased fungal burden and elevated mortality risk in cryptococcal meningitis patients (Jarvis et al. 2014). Thus, classifying patients based on CrAg titers could offer prognostic value, identifying those requiring a lumbar puncture versus those treatable with fluconazole alone. This enables personalized diagnostic and treatment strategies for individuals with cryptococcosis.

**Fig. 1:**
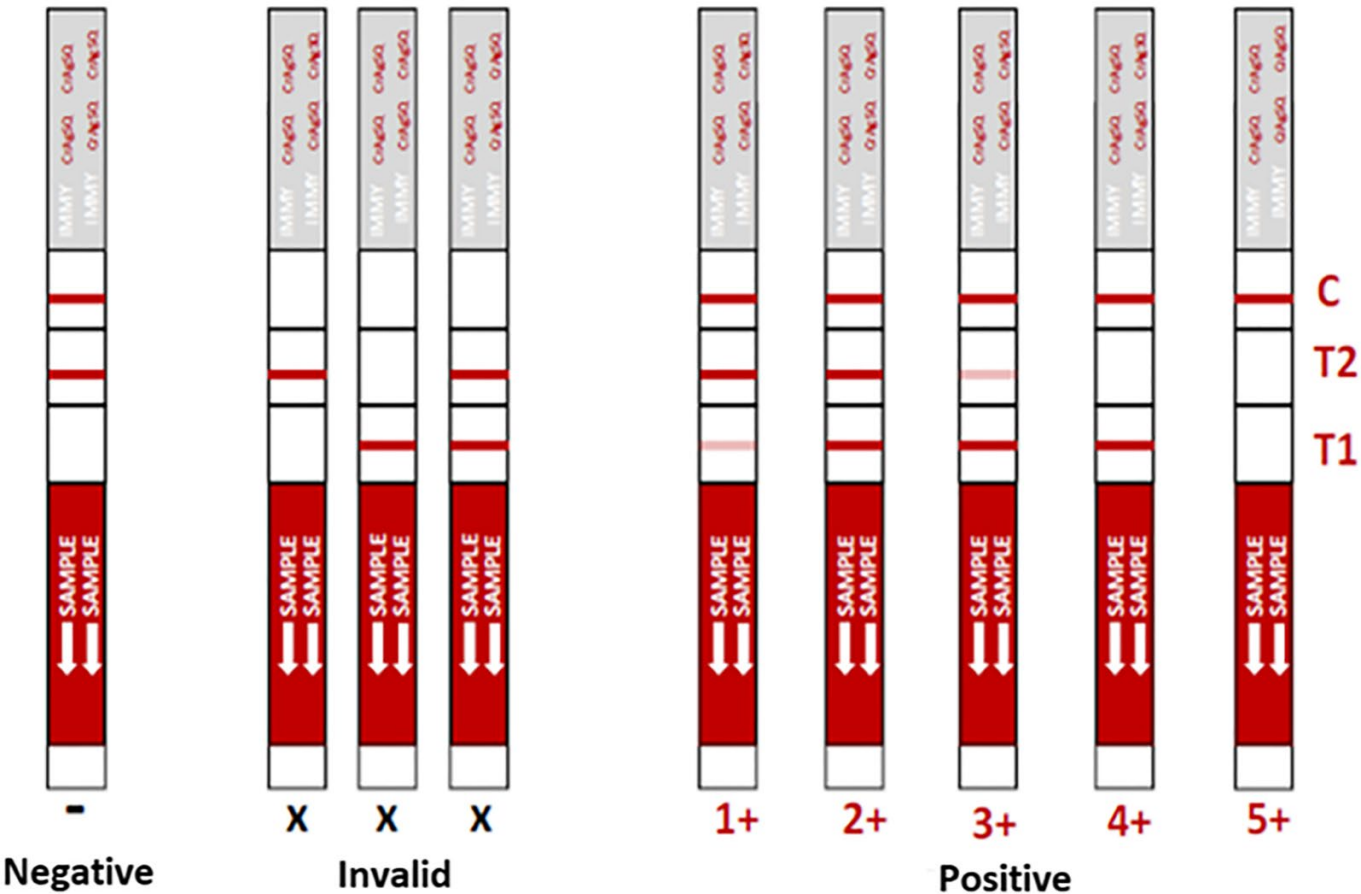
Interpretation of Cryptococcal Antigen Semi-Quantitative (CrAgSQ) lateral flow assay (LFA) IMMY test results. The "C" band indicates the internal control of the test, which must have visible coloring to validate a result. The absence of the T1 band in the presence of T2 and C indicates a negative test result. The test is positive when T2 > T1, T2 = T1 and T2 < T1, with values of 1 +, 2 + and 3 +, respectively. When there is presence of T1 in the absence of T2, the value is 4 +, and when there is absence of both T1 and T2, the value is 5 +. Invalid test is considered when only a T2 and/or a T1 band appears (without control line)

The introduction of the new cryptococcal antigen semi-quantitative (CrAgSQ) LFA could provide a better assessment of the risk and resolves the limitations of the qualitative LFA, as it can classify patients into five stages that correlate with the severity of cryptococcosis (Blasich et al. 2021; Tadeo et al. 2021) as depicted in Fig. 1**.** However, interpreting the test is complex and requires intensive and specialized training for healthcare workers using it, which is difficult and time-consuming. Other factors, such as the number of weekly tests performed in the health facility, the turnover of untrained technicians responsible for the specific diagnostic technique as well as the visual acuity of the observer, can also impact the reading reproducibility of the strip.

The development of mobile applications that employ artificial intelligence to interpret point-of-care tests (POCTs), such as LFAs for diagnosing cryptococcosis, is now feasible due to the adaptability and widespread distribution of smartphones (Roda et al. 2016; Xu et al. 2015). This innovative approach not only enhances accessibility but also holds the promise of standardizing and objectivizing test result interpretations, reducing the impact of human subjectivity and interobserver variability, and ultimately improving the reliability and consistency of diagnostic outcomes. A previous proof of concept has already demonstrated the feasibility of an interpretation app for IMMY's CrAg® LFA qualitative test, suggesting similar success with other LFA tests like the CrAgSQ LFA (Bermejo-Peláez et al. 2023).

The primary aim of this study is to introduce a digital platform based on artificial intelligence for the automated interpretation of the CrAgSQ LFA. This test is used for one-step detection and semi-quantification of *Cryptococcus* antigen, streamlining the diagnosis process and enhancing accuracy.

## Materials and methods

### Study samples and data collection

A total of fifty-three *Cryptococcus* antigen concentrations, spanning from 0 to 5,000 ng/ml, were prepared from a stock solution provided by the manufacturer (IMMY) and subjected to analysis using the CrAgSQ LFA (IMMY, OK, USA). These concentrations were prepared and evaluated over the course of three separate days. The antigen dilutions were carried out in standardized human sera sourced from Merck, Sigma-Aldrich, Madrid, Spain. For each concentration, two distinct LFA strips were inoculated on the same day, and these were also subjected to duplicate testing over the span of three different days. The testing procedures were conducted for each of the fifty-three sequential concentrations in strict accordance with the manufacturer's recommended protocols.

For the purpose of assessing variability, each LFA strip was digitally captured and photographed twice using two different smartphone models with varying technical capabilities. The first smartphone model used was the Motorola Moto E6, categorized as low-mid range, while the second was the Samsung Galaxy S9, categorized as upper-mid range. Both devices featured a camera with a resolution of ≥ 12 MPx. All LFAs were photographed using the TiraSpot mobile app (Spotlab, Madrid, Spain), which features a mask specifically adjusted to the size and shape of the CrAgSQ test strip, ensuring precise and standardized image capture. All images and relevant sample information were uploaded to a cloud-based platform for further analysis.

Additionally, each LFA strip was visually read according to the manufacturer's instructions by two independent trained observers who were blinded to the CrAg concentration. Both observers read the strips independently and recorded their scored results in a database. The interpretation of the images was conducted at a different facility, where the results were subsequently scored. These scores were then compared, and any discrepancies were noted.

To ensure an objective comparison between visual observations and AI readings, the study design included an independent observer responsible for addressing any discrepancies between the initial visual readings and the AI interpretations. This predefined process aimed to maintain objectivity, enhance transparency, minimize potential bias, and uphold data integrity during the evaluation of AI performance.

### Artificial intelligence algorithm for automatic reading of CrAgSQ LFA

**Fig. 2:**
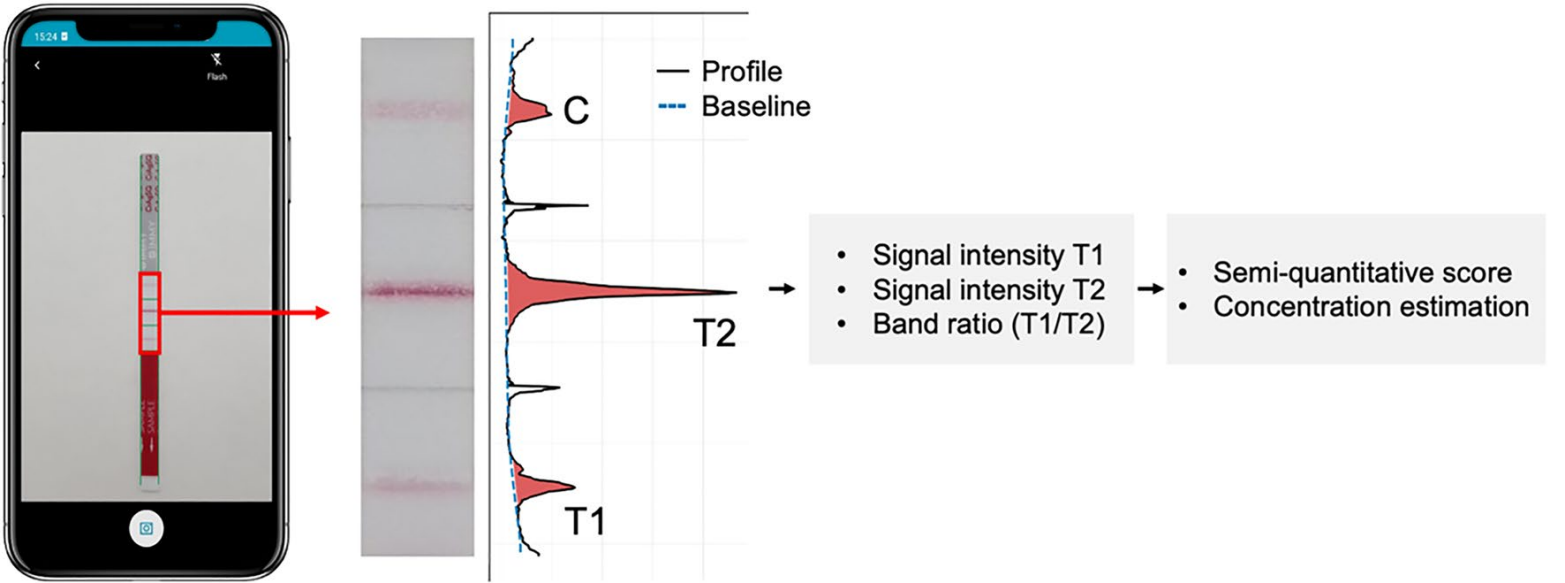
Workflow of the proposed system to automatically read the Cryptococcal Antigen Semi-Quantitative (CrAgSQ) lateral flow assay (LFA). First, the LFA is digitized through a mobile application. Then, the image is processed by cropping the region of interest, where the line signal intensities are quantified to determine the corresponding semi-quantitative score or even estimate the cryptococcal antigen (CrAg) concentration

An AI algorithm for automatic reading of the CrAgSQ LFA was developed following a specific processing pipeline (Fig. 2). Initially, the image captured by the mobile app undergoes preprocessing using a color correction algorithm. This correction enhances the algorithm's robustness against variations caused by different illumination scenarios, such as warm or cool lighting, which can introduce variations in pixel intensity. The color correction algorithm employs a gray world color normalization method, which assumes that the average color in a scene should be neutral gray. This method adjusts the colors by scaling the all image channels (red, green, and blue) based on the mean values of each channel, ensuring consistent color representation across different lighting conditions. These enhancements in image preprocessing ensure that the subsequent AI interpretations are reliable and consistent, even under varied environmental conditions. Once preprocessed, the image is subjected to an automatic method based on computer vision that accurately crops the region of interest, focusing only on the area containing the control and test bands, while discarding irrelevant portions of the image.

Subsequently, the cropped image is converted into grayscale, and a one-dimensional signal profile is generated by calculating the average intensity along the short axis of the resulting image. To account for different ambient lighting conditions and potential image imperfections like uneven illuminations or shadows, the algorithm estimates and corrects the background of the signal profile.

Finally, the intensities of the control and test lines (C, T1, and T2) are quantified based on the corrected signal profile. An automatic scoring system is implemented, able to assign each LFA a score as negative, 1 +, 2 +, 3 +, 4 +, or 5 +. The scoring calculation is derived from the manufacturer's instructions and follows these rules: if the intensity of test band T1 is lower than that of test band T2, the score is designated as 1 +. When the intensities of T1 and T2 are equal (considered when they are within a ± 20% difference), the score is recorded as 2 +. Conversely, if the intensity of T1 is higher than that of T2, the score is assigned as 3 +. If only the T1 band is present without T2, the score is designated as 4 +. In cases where only the control band (C) is detected, the score is recorded as 5 +. If only the T2 band and the control band are visible, the interpretation is considered negative. The scoring process is carried out automatically, enabling efficient and standardized analysis of LFAs based on the quantified intensities of the test bands.

Additionally, to further explore the relationship between the quantification of the test lines and the concentration the ratio between the intensities of T1 and T2 was calculated. This ratio measurement offers the potential for a quantitative, rather than semi-quantitative, interpretation of the LFA and explores whether the algorithm is capable of calculating the actual value of the antigen concentration.

It should be noted that the implemented AI model in this study, intentionally designed to be free from the need for a training process, ensures a lack of bias, and all images collected in the study, as outlined in the preceding section, can be directly utilized to evaluate the model's performance.

### Statistical analysis

To assess the agreement between visual and AI readings of CrAgSQ LFAs, we employed a confusion matrix, providing a comprehensive view of true positives, true negatives, false positives, and false negatives for each CrAgSQ score. Additionally, the interquartile range (IQR) was employed as a robust measure to evaluate the variability within the concentration range associated with CrAgSQ scores. For the purpose of evaluating the regression analysis that relates signal intensity to CrAg concentration, Pearson correlation (r) was calculated. This analysis was carried out utilizing GraphPad Prism 10.0 (GraphPad Software, Boston, Massachusetts USA) to construct the regression model. To compare the frequency of inconsistencies committed by visual and AI readings and assess the reliability in result interpretation, we conducted a chi-square test and considered a p-value < 0.05 as statistically significant between both reading methods.

## Results

### Comparison between automatic and visual interpretation of CrAgSQ LFA

**Fig. 3:**
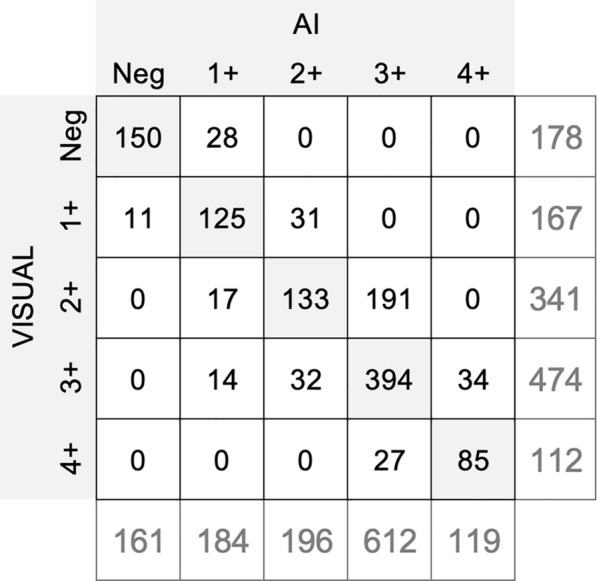
Confusion matrix comparing visual and AI-based readings of the Cryptococcal Antigen Semi-Quantitative (CrAgSQ) lateral flow assay (LFA)

Overall, a total of 318 CrAgSQ LFA strips were photographed and visually interpreted, resulting in 1,272 photos which were further analyzed by the proposed algorithm, assigning a semi-quantitative score to each strip image. A comparison was made between the automatic readings from the algorithm and the visual readings performed by the observers. In this comparison, 386 images (30%) exhibited discrepancies between the algorithm and observer readings. The confusion matrix between the algorithm's automatic readings and the visual readings by the observers is shown in Fig. 3. The confusion matrices that compare visual and AI results for each mobile phone model separately, as well as the one comparing AI results across the two mobile phone models, are displayed in supplementary Figure S1.

According to Fig. 3, observers classified 11 CrAgSQ LFA strips (CrAg concentrations from 2 to 6 ng/ml) as 1 + whilst the algorithm interpreted all negative. An independent observer classified 8 out of 11 as negative in a posterior analysis and finally only 3 were truly discrepant corresponding to antigen concentrations of 5–6 ng/ml. Additionally, there were 28 discrepancies between the algorithm's 1 + and the observer's negative scores (CrAg concentrations ranging from 0.25 to 10 ng/ml), being attributed to the algorithm detecting low T1 band intensities that might go unnoticed by the human eye.

Observers classified 17 cases as 2 + , whereas the algorithm interpreted them as 1 + (CrAg concentration ranging from 20 to 30 ng/ml). On the other hand, 31 images were visually classified as 1 + while the algorithm assigned 2 + (CrAg concentrations ranging from 25 to 50 ng/ml). The algorithm's quantitative analysis can capture differences in intensity that might not be evident to the naked eye, being capable of discerning when the T1 band intensity is actually lower than the T2 band, or when the bands indeed have similar intensity (within a margin of 20%, as explained in Sect. "Artificial intelligence algorithm for automatic reading of CrAgSQ LFA").

Similar results can be observed when analyzing discrepancies between 2 + and 3 + scores. Thirty-two images were initially categorized as 3 + by observers but interpreted as 2 + by the algorithm (CrAg concentrations ranging from 30 to 70 ng/ml). One hundred and ninety-one visual readings were designated as 2 + by observers, while the algorithm identified them as 3 + (CrAg concentration ranging from 50 to 145 ng/ml).

There were 27 discrepancies between the algorithm's assigned scores of 3 + and the observers' scores of 4 + (CrAg concentrations ranging from 210 to 5000 ng/ml). The algorithm was capable of detecting very subtle T2 lines that escaped the notice of the human observers in these instances. Among the 34 cases where the algorithm assigned a 4 + while the observers scored a 3 + , an independent visual examination of the images unveiled that 12 of them were indeed 4 + (without a T2 band), as correctly identified by the algorithm.

Another noteworthy observation was 14 discrepancies where the algorithm assigned a 1 + score while observers interpreted them as 3 + (CrAg concentrations ranging from 15 to 30 ng/ml). Subsequent independent analysis confirmed the accuracy of the algorithm in identifying them as 3 + scores, with the T2 band intensity exceeding that of T1.

### Reading reliability

All LFA strips inoculated only with human sera to be used as controls were identified correctly as negative by both observers and AI algorithm.

**Fig. 4:**
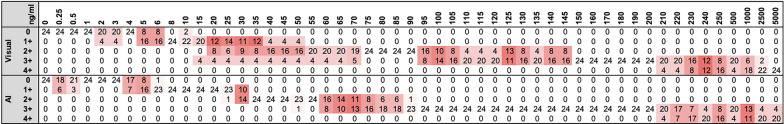
Number of readings (24 for each concentration: 2 replicates × 3 days × 2 observers × 2 photos) for each semi-quantitative score grouped by concentration, for both visual (upper rows) and AI-based (lower rows) interpretations. Discrepancies in LFA readings are highlighted in red

As derived from Fig. 4, the AI algorithm tends to generate more positive results in comparison to visual interpretation indicating a slightly higher sensitivity of the test when it is read by AI. At lower concentrations, specifically within the range of 0.25 to 0.5 ng/ml, all observers consistently interpreted the results as negative. However, the AI, on some occasions, identified certain associated photographs as positive, assigning them a score of 1 +. Conversely, when dealing with concentrations ranging from 2 to 3 ng/ml, the AI uniformly categorized all images as negative, while human observers occasionally assigned a positive score of 1 +.

To evaluate the consistency and reliability of the readings, we independently calculated the number of LFAs that exhibited inconsistent results for both visual observations and AI readings. As detailed in the methods section, each CrAgSQ LFA underwent four separate photographic captures, with two of them taken using each of the two smartphone models employed. For every instance of LFA picture, visual interpretations of the LFA results were conducted, resulting in four readings in total, with each reading being performed by two different observers. In this context, discrepancies were defined as situations where, in at least one of the four interpretations, there was a difference compared to the others.

Out of 318 LFAs, 100 (31.4%) exhibited discrepancies in the visual readings conducted by the observers. In contrast, only 35 (11%) LFAs exhibited discrepancies in the AI interpretations derived from the four different photos. Discrepancies in this context were defined as cases where at least one of the four AI interpretations differed from the others. These findings indicate that the AI-based approach showed higher reading consistency (fewer discrepancies) compared to visual assessments (p-value < 0.0001).

We conducted an additional assessment to evaluate the consistency by categorizing LFA readings according to CrAg concentration as shown in Fig. 4. This assessment revealed that visual readings categorized 36 (68%) concentrations differently out of the 53 different concentrations tested, whereas AI readings displayed inconsistencies in 24 (45%) of these concentrations. Furthermore, visual readings spanned across three categories (ranging from 1 + to 3 +) for certain concentrations, but this phenomenon did not occur in AI scoring as depicted in Fig. 4.

### Relationship between semi-quantitative score and concentration

**Fig. 5:**
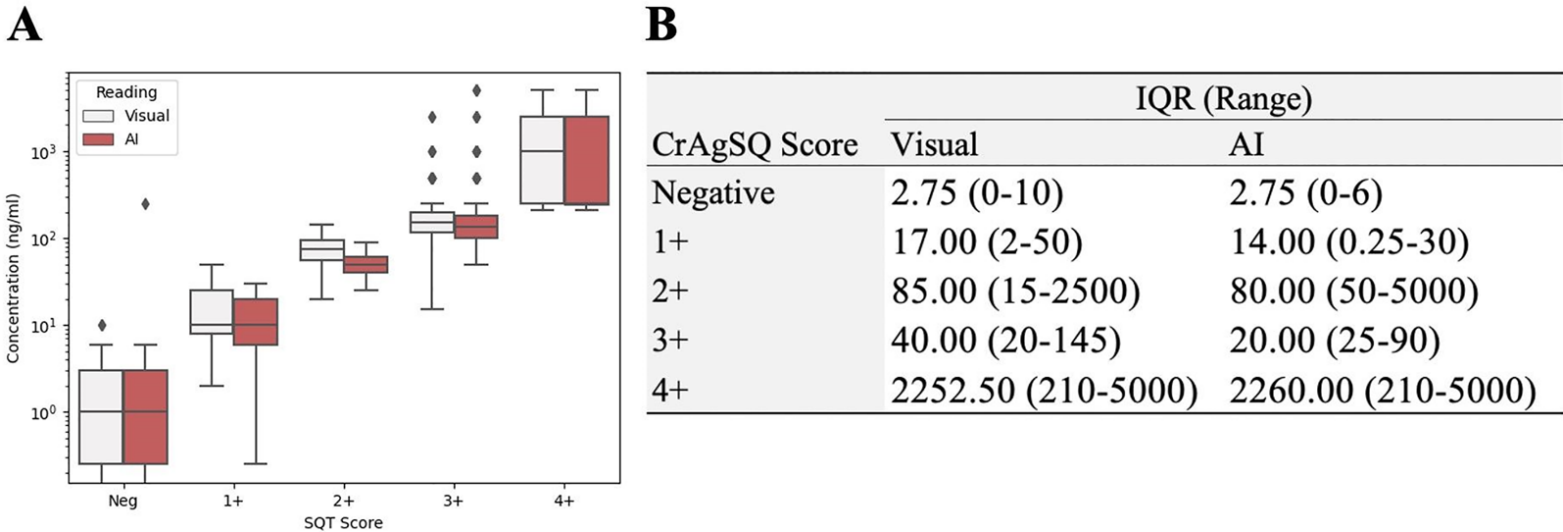
**A** Box plot comparison between visual and AI-based readings with corresponding cryptococcal antigen (CrAg) concentration ranges. **B** Interquartile ranges (IQR) and concentration ranges by Cryptococcal Antigen Semi-Quantitative (CrAgSQ) score for both visualization and AI readings

Figure 5A shows the concentration range for each of the semiquantitative scores, for both visual and AI-based readings. Figure 5B presents the interquartile ranges (IQR) of concentrations grouped by each CrAgSQ score for both visual and AI-based readings independently. The results demonstrate that the AI-based readings exhibit distinct and well-defined distribution ranges of CrAg concentrations for each semi-quantitative score.

Moreover, as derived from Fig. 5, the variability in concentration range associated with visual interpretation was in general higher than the one obtained by the AI. This finding indicates that the AI-based approach offers a more consistent correlation between semi-quantitative scores and the actual concentration of CrAg. Notably, this enhanced consistency holds particular relevance for scores 2 + and 3 + in AI assessments, aligning with previous studies that underscore the critical nature of the threshold between 2 + and 3 + in predicting cryptococcal meningitis (Blasich et al. 2021; Tadeo et al. 2021).

### Relationship between signal intensity and concentration

**Fig. 6:**
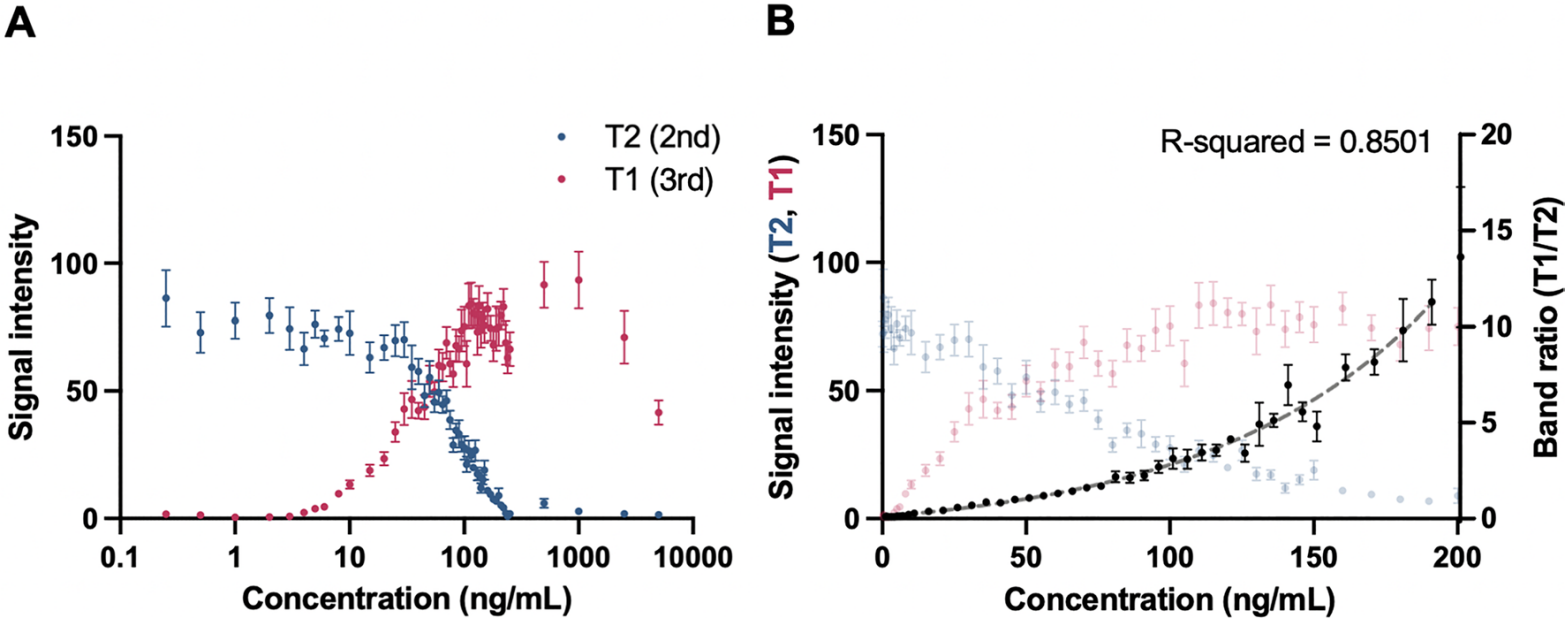
**A** Dose–response curve of the Cryptococcal Antigen Semi-Quantitative (CrAgSQ) lateral flow assay (LFA) for both T1 and T2 lines, revealing the presence of the hook effect for T1 line after approximately 190 ng/ml. **B** Quantitative results and regression analysis curve depicting the relationship between cryptococcal antigen (CrAg) concentration and band ratio signal intensity (T1/T2)

Signal intensities of both T1 and T2 lines were quantified for each CrAgSQ LFA. Figure 6A illustrates the dose–response curve, showcasing the variation of signal intensity with changes in CrAg concentration. It should be noted that with concentrations greater than 190 ng/ml, the appearance of the postzone effect (or high-dose hook effect) becomes evident. This effect leads to a contradictory decrease in the T1 test line intensity as the concentration of the antigen increases. The presence of the postzone effect beyond 190 ng/ml introduces limitations to establishing a direct relationship between the ratio of T1 to T2. Consequently, the dynamic range for which such a relationship can be reliably established lies between 0 and 190 ng/ml. In Fig. 6B, the signal band ratio (T1/T2) is displayed within the dynamic range, along with the fitted curve using the 4PL regression model, representing the relationship between the band ratio and concentration within this range. A Pearson correlation coefficient of 0.85 was found, demonstrating a strong relationship between the band ratio (T1/T2) quantified by the AI and the corresponding CrAg concentration.

## Discussion and conclusions

Cryptococcosis remains a significant global health concern, particularly for immunocompromised individuals. Timely and accurate diagnosis is critical to initiate prompt and appropriate treatment, reducing morbidity and mortality rates associated with the disease. The use of point-of-care devices such as lateral flow assays provides a rapid and accessible approach for diagnosing various diseases, offering timely results, and simplifying healthcare delivery, particularly in resource-limited settings. In this study, we have presented an innovative AI algorithm designed to automatically interpret CrAgSQ LFA in a fully automated and objective manner. Leveraging a mobile application, the algorithm processes a single photo, eliminating the need for manual visual inspection. The results demonstrate the algorithm's ability to accurately quantify the signal intensities of the test bands, leading to the assignment of semi-quantitative scores. Our findings highlight the algorithm's proficiency in providing reliable and consistent readings, reducing the impact of human subjectivity and interobserver variability.

According to two previous studies, a key threshold for the CrAgSQ is 2 + because all patients with a CrAgSQ score of 3 + or higher, have cryptococcal meningitis, despite some of them being classified as asymptomatic or paucisymptomatic (Blasich et al. 2021; Tadeo et al. 2021). The results of the study, based on the evaluation of visual and AI-based interpretations, underscore the improved accuracy and reduced variability exhibited by the algorithm when distinguishing between CrAgSQ scores of 2 + and 3 +. Notably, visual observers demonstrated discrepancies across a broad concentration range spanning from 15 to 145 ng/ml in this critical score distinction. In contrast, the AI algorithm displayed discrepancies only within the narrow concentration range of 60–90 ng/ml, thus demonstrating superior capability and consistency in the differentiation between CrAgSQ scores of 2 + and 3 +. Furthermore, the AI algorithm achieved a narrower range of CrAg concentrations (25–90 ng/ml) for 2 + readings compared to visual assessments (20–145 ng/ml), enhancing the precision of patient risk assessment. This wide variability in visual interpretation can be attributed to the inherent difficulty in visually comparing signal intensities quantitatively.

Furthermore, we have demonstrated the capability of predicting CrAg concentrations solely based on a picture of the LFA, which can serve as a validation for the score results obtained through visual or AI interpretation. Through an analysis of the ratio of test line intensities, a robust correlation with CrAg concentration was observed, with a Pearson correlation coefficient of 0.85, within the range of 0–190 ng/ml. According to the results of the algorithm presented in Fig. 4, a + 3 score reading begins at an antigen concentration of 60 ng/ml. In the range of 60–85 ng/ml antigen concentrations, the algorithm assigns scores of 2 + and 3 + almost interchangeably. However, at concentrations of ≥ 90 ng/ml, nearly all results, except one, indicate a score of 3 +. It is important to note that beyond 190 ng/ml, the presence of the postzone effect complicates the direct quantification of CrAg concentration based on signal intensity measurement. Immunoassays rely on the formation of antigen–antibody complexes to produce a visible or measurable signal. The optimal ratio of antigen to antibody is crucial for these reactions to occur efficiently. When there is an excessive amount of antigen present, it can saturate all available binding sites on the antibodies. This prevents the formation of the large antigen–antibody complexes necessary for a visible reaction. As a result, despite a high concentration of antigen, the expected agglutination or precipitation does not occur, leading to a false-negative result. In such cases, the appearance of a very faint T2 band or its disappearance, indicating a + 4 score, renders signal intensity quantification unnecessary. Consequently, strategies such as sample dilution, which have been employed with the qualitative CrAg test (Tadeo et al. 2021), are effectively addressed with the CrAgSQ test. Furthermore, the score information is reinforced with the caution that if the result indicates an antigen concentration of ≥ 60 ng/ml, an extensive patient evaluation should be conducted.

The study was done in a controlled laboratory environment, which may not fully reflect the real-world conditions and challenges encountered in various clinical settings. In this context, no 5 + scores were observed in our dataset, even when using high CrAg concentrations (5000 ng/ml). Notably, similar findings were reported in a separate study involving clinical samples (Blasich et al. 2021), where they also did not encounter any 5 + scores. However, it is worth highlighting that in another study conducted on clinical samples, the authors did observe a small percentage of cases (2%) with 5 + scores (Tadeo et al. 2021).

Future work will involve further assessments of this approach by means of a clinical study with patients with and without cryptococcal meningitis diagnosed by means of gold-standard techniques.

The integration of AI technology in the interpretation of CrAgSQ LFAs offers distinct advantages, as it enables an automatic and objective analysis relying solely on a photo taken with a smartphone. The result obtained by the AI algorithm can overcome the issue of extensive training before its implementation in the clinical setting, allowing for easier risk assessment of every HIV-positive patient at risk of cryptococcosis. Additionally, it can decrease the cost and the risk of universal lumbar puncture for every patient with a positive CrAg test. It is well-known that many patients reject the lumbar puncture procedure, and in many settings, the necessary devices for this invasive procedure may not be readily available. Therefore, a more accurate risk assessment provided by the AI algorithm would be extremely helpful for the patient and the healthcare system. In addition, the results are recorded in the cloud which facilitates the estimation of the burden of the disease as well as active surveillance and epidemiological studies.

This technology holds the potential for easy adaptability to interpret LFAs for various diseases and can be deployable at the edge, enabling real-time and on-site interpretation, revolutionizing point-of-care diagnostics and advancing healthcare accessibility worldwide.

### Supplementary Information

Supplementary Material 1.

## Data Availability

The data supporting the results of this study will be made available upon reasonable request from the corresponding author.

## Abbreviations

AI: Artificial Intelligence
CrAg: Cryptococcal Antigen
CrAg SQ: Cryptococcal Antigen Semi-Quantitative
LFA: Lateral Flow Assay
POCT: Point Of Care Test
WHO: World Health Organization

## References

[CR1] Antinori S (2013) New insights into HIV/AIDS-associated cryptococcosis. ISRN AIDS 2013:1–22. 10.1155/2013/471363 10.1155/2013/471363 PMC3767198 24052889

[CR2] Bermejo-Peláez D, Medina N, Álamo E, Soto-Debran JC, Bonilla O, Luengo-Oroz M, Rodriguez-Tudela JL, Alastruey-Izquierdo A (2023) Digital platform for automatic qualitative and quantitative reading of a cryptococcal antigen point-of-care assay leveraging smartphones and artificial intelligence. J Fung 9(2):217. 10.3390/jof9020217 10.3390/jof9020217 PMC9961444 36836331

[CR3] Blasich NP, Wake RM, Rukasha I, Prince Y, Govender NP (2021) Association of semi-quantitative cryptococcal antigen results in plasma with subclinical cryptococcal meningitis and mortality among patients with advanced HIV disease. Med Mycol 59(10):1041–1047. 10.1093/mmy/myab038 34169984 10.1093/mmy/myab038 PMC8487765

[CR4] Firacative C, Lizarazo J, Illnait-Zaragozí MT, Castañeda E (2018) The status of cryptococcosis in Latin America. Mem Inst Oswaldo Cruz. 10.1590/0074-02760170554 29641639 10.1590/0074-02760170554 PMC5888000

[CR5] Firacative C, Trilles L, Meyer W (2021) Recent advances in cryptococcus and cryptococcosis. Microorganisms 10(1):13. 10.3390/microorganisms10010013 35056462 10.3390/microorganisms10010013 PMC8779235

[CR6] Jarvis JN, Bicanic T, Loyse A, Namarika D, Jackson A, Nussbaum JC, Longley N, Muzoora C, Phulusa J, Taseera K, Kanyembe C, Wilson D, Hosseinipour MC, Brouwer AE, Limmathurotsakul D, White N, Van Der Horst C, Wood R, Meintjes G, Harrison T (2014) Determinants of mortality in a combined cohort of 501 patients with HIV-associated cryptococcal meningitis: implications for improving outcomes. Clin Infect Dis 58(5):736–745. 10.1093/cid/cit794 24319084 10.1093/cid/cit794 PMC3922213

[CR7] Medina N, Alastruey-Izquierdo A, Bonilla O, Gamboa O, Mercado D, Pérez JC, Salazar LR, Arathoon E, Denning DW, Rodriguez-Tudela JL (2021) A rapid screening program for histoplasmosis, tuberculosis, and cryptococcosis reduces mortality in HIV patients from guatemala. J Fung 7(4):268. 10.3390/jof7040268 10.3390/jof7040268 PMC8065950 33916153

[CR8] Rajasingham R, Govender NP, Jordan A, Loyse A, Shroufi A, Denning DW, Meya DB, Chiller TM, Boulware DR (2022) The global burden of HIV-associated cryptococcal infection in adults in 2020: a modelling analysis. Lancet Infect Dis 22(12):1748–1755. 10.1016/S1473-3099(22)00499-6 36049486 10.1016/S1473-3099(22)00499-6 PMC9701154

[CR9] Roda A, Michelini E, Zangheri M, Di Fusco M, Calabria D, Simoni P (2016) Smartphone-based biosensors: a critical review and perspectives. TrAC Trends Anal Chem 79:317–325. 10.1016/j.trac.2015.10.019 10.1016/j.trac.2015.10.019

[CR10] Tadeo KK, Nimwesiga A, Kwizera R, Apeduno L, Martyn E, Okirwoth M, Nalintya E, Rajasingham R, Williams DA, Rhein J, Meya DB, Kafufu B, Boulware DR, Skipper CP (2021) Evaluation of the diagnostic performance of a semiquantitative cryptococcal antigen point-of-care assay among HIV-infected persons with cryptococcal meningitis. J Clin Microbiol 59(8):e00860-e921. 10.1128/JCM.00860-21 34076472 10.1128/JCM.00860-21 PMC8373252

[CR11] Tang MW, Clemons KV, Katzenstein DA, Stevens DA (2016) The cryptococcal antigen lateral flow assay: a point-of-care diagnostic at an opportune time. Crit Rev Microbiol 42(4):634–642. 10.3109/1040841X.2014.982509 25612826 10.3109/1040841X.2014.982509

[CR12] World Health Organization (2018) Guidelines for the diagnosis, prevention and management of cryptococcal disease in HIV-infected adults, adolescents and children 26110194

[CR13] World Health Organization (2022a) Guidelines for diagnosing, preventing and managing cryptococcal disease among adults, adolescents and children living with HIV. World Health Organization 35797432

[CR14] World Health Organization (2022b) WHO fungal priority pathogens list to guide research, development and public health action. World Health Organization. https://apps.who.int/iris/handle/10665/363682

[CR15] Xu X, Akay A, Wei H, Wang S, Pingguan-Murphy B, Erlandsson B-E, Li X, Lee W, Hu J, Wang L, Xu F (2015) Advances in smartphone-based point-of-care diagnostics. Proc IEEE 103(2):236–247. 10.1109/JPROC.2014.2378776 10.1109/JPROC.2014.2378776

