# Supplementary material for: Artificial intelligence-driven mobile interpretation of a semi-quantitative cryptococcal antigen lateral flow assay
Source: IMA Fungus. 2024 Aug 30;15:27. doi: 10.1186/s43008-024-00158-5 (PMC11365246; doi:10.1186/s43008-024-00158-5)
Supplement: Supplementary file 1 — Supplementary Material 1. [file 43008_2024_158_MOESM1_ESM.docx]

**Supplementary information**

**
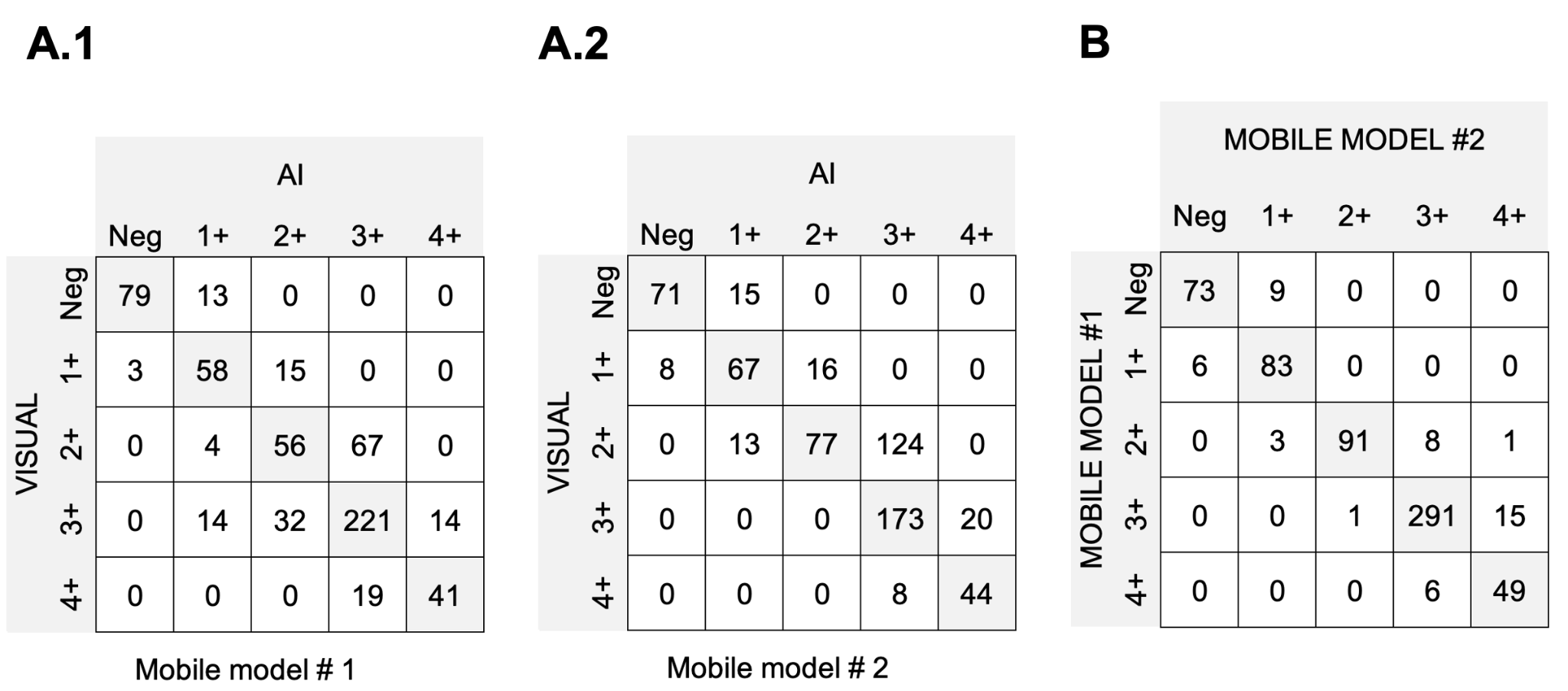
**

**Supplementary Figure S1. A.1-A2:** Confusion matrices comparing visual and AI-based Cryptococcal Antigen Semi-Quantitative (CrAgSQ) interpretation separated by each of the two smartphone models used in the study. **B**: Confusion matrix comparing AI-based interpretations across the two smartphone models.
